# Supplementary material for: Active site recovery and N-N bond breakage during hydrazine oxidation boosting the electrochemical hydrogen production
Source: Nat Commun. 2023 Apr 10;14:1997. doi: 10.1038/s41467-023-37618-2 (PMC10083172; doi:10.1038/s41467-023-37618-2)
Supplement: Supplementary file 1 — Supporting Information [file 41467_2023_37618_MOESM1_ESM.pdf]

Supporting Information

Active site recovery and N-N bond breakage during hydrazine oxidation boosting the electrochemical hydrogen production

Libo Zhu[1,2#], Jian Huang[1#], Ge Meng[1,2], Tiantian Wu[1], Chang Chen[1,2], Han Tian[1], Yafeng Chen[3], Fantao Kong[1], Ziwei Chang[4], Xiangzhi Cui[1,2,5*], Jianlin Shi[1*]

[1] State Key Lab of High Performance Ceramics and Superfine Microstructure, Shanghai Institute of Ceramics, Chinese Academy of Sciences, Shanghai 200050, PR China.

[2] Center of Materials Science and Optoelectronics Engineering, University of Chinese Academy of Sciences, Beijing 100049, PR China.

[3] Collaborative Innovation Center of Steel Technology, University of Science and Technology Beijing, Beijing 100083, PR China.

[4] School of Physical Science and Technology, Shanghai Tech University, Shanghai 201210, PR China.

[5] School of Chemistry and Materials Science, Hangzhou Institute for Advanced Study, University of Chinese Academy of Sciences, Hangzhou 310024, PR China.

[#]These authors contributed equally to this manuscript: Libo Zhu, Jian Huang.

[*]Email: jlshi@mail.sic.ac.cn (J. Shi), cuixz@mail.sic.ac.cn (X. Cui).

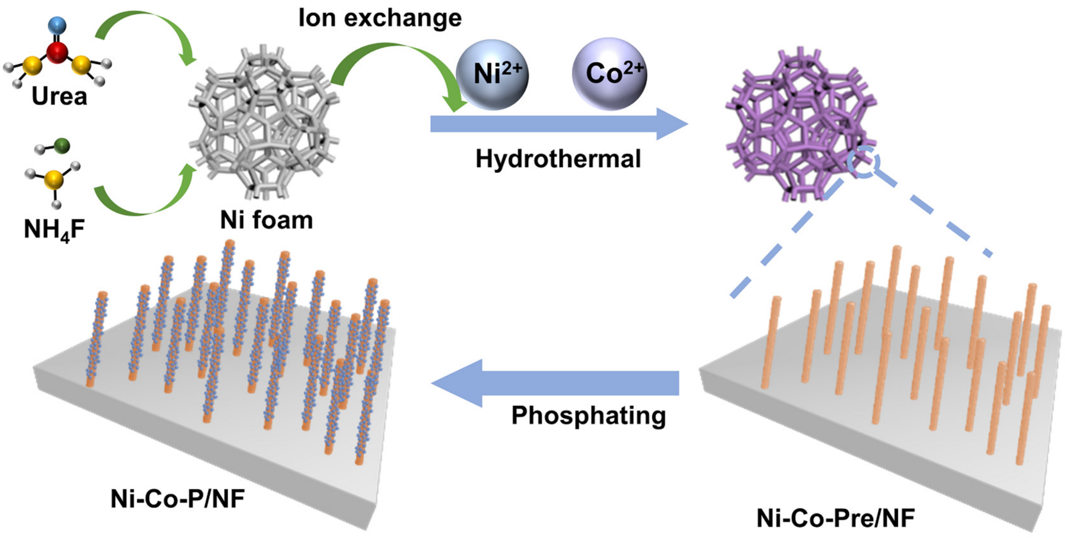

Fig. S1. Schematic illustration of synthesis of Ni-Co-P/NF.

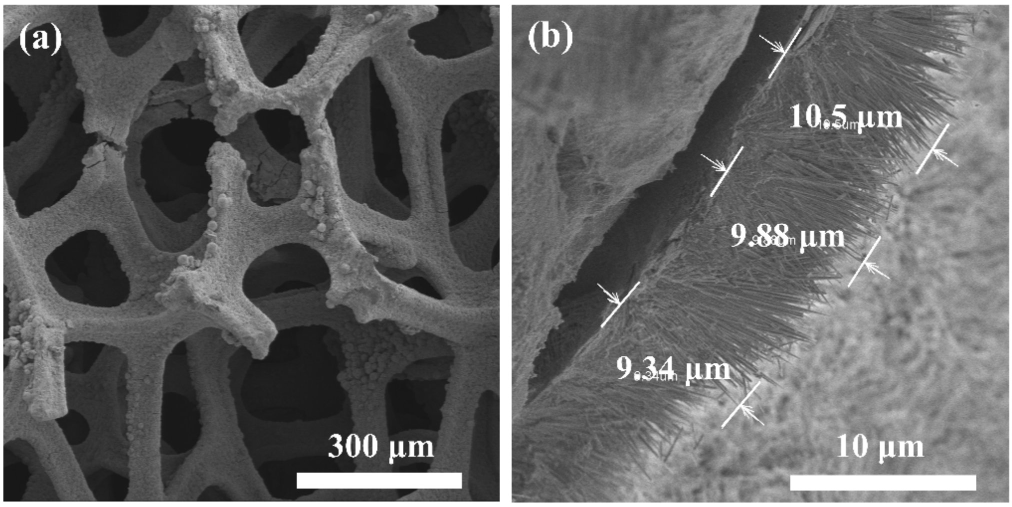

Fig. S2. Typical FESEM images at different magnifications. (a) Ni-Co-Pre/NF; (b) Thickness of nanowire arrays on the surface of Ni-Co-Pre/NF.

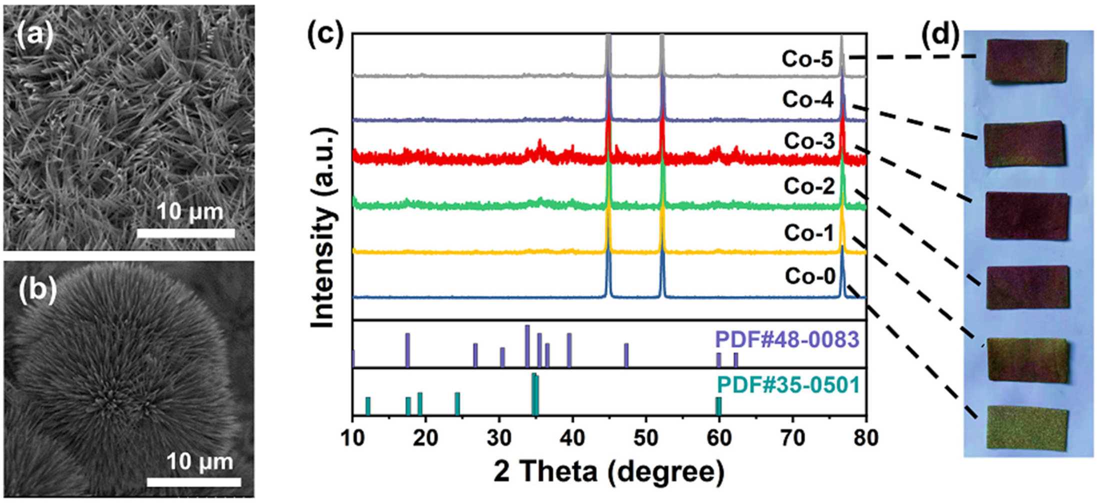

Fig. S3. Typical FESEM of Ni-Co-Pre/NF with (a) Co-3, (b) Co-5; (c) XRD patterns of precursors with different Co source addition amount (PDF#48-0083: $Co(CO_3)_{0.5}(OH)$; PDF#35-0501: $Ni(CO_3)_{0.5}(OH)$), and the corresponding Digital photographs (d).

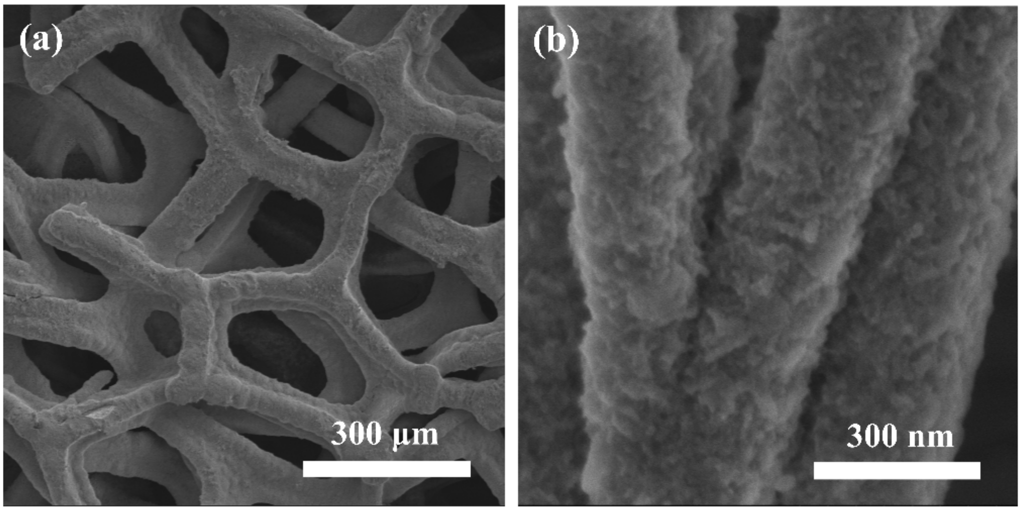

Fig. S4. Typical FESEM images of Ni-Co-P/NF (a, b) at different magnifications.

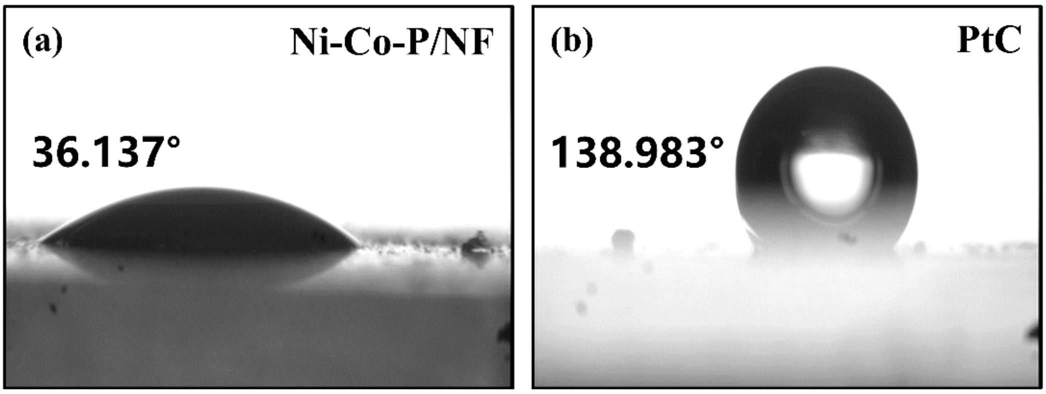

Fig. S5. The images of water contact angle measurements. (a) Ni-Co-P/NF; (b) Pt/C.

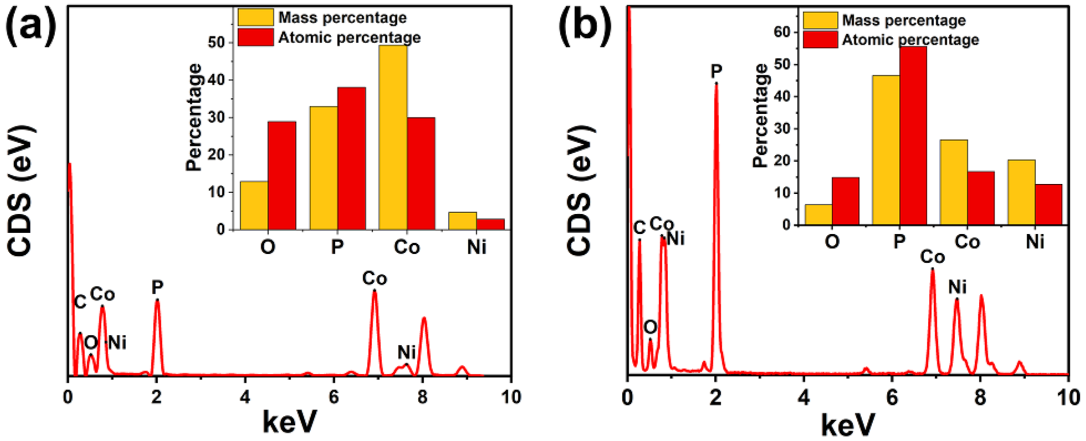

Fig. S6. TEM-EDS images of Ni-Co-P/NF of different areas in Fig. 1c. (a) Area 1; (b) Area 2, with the mass percentage and atomic percentage in the inset.

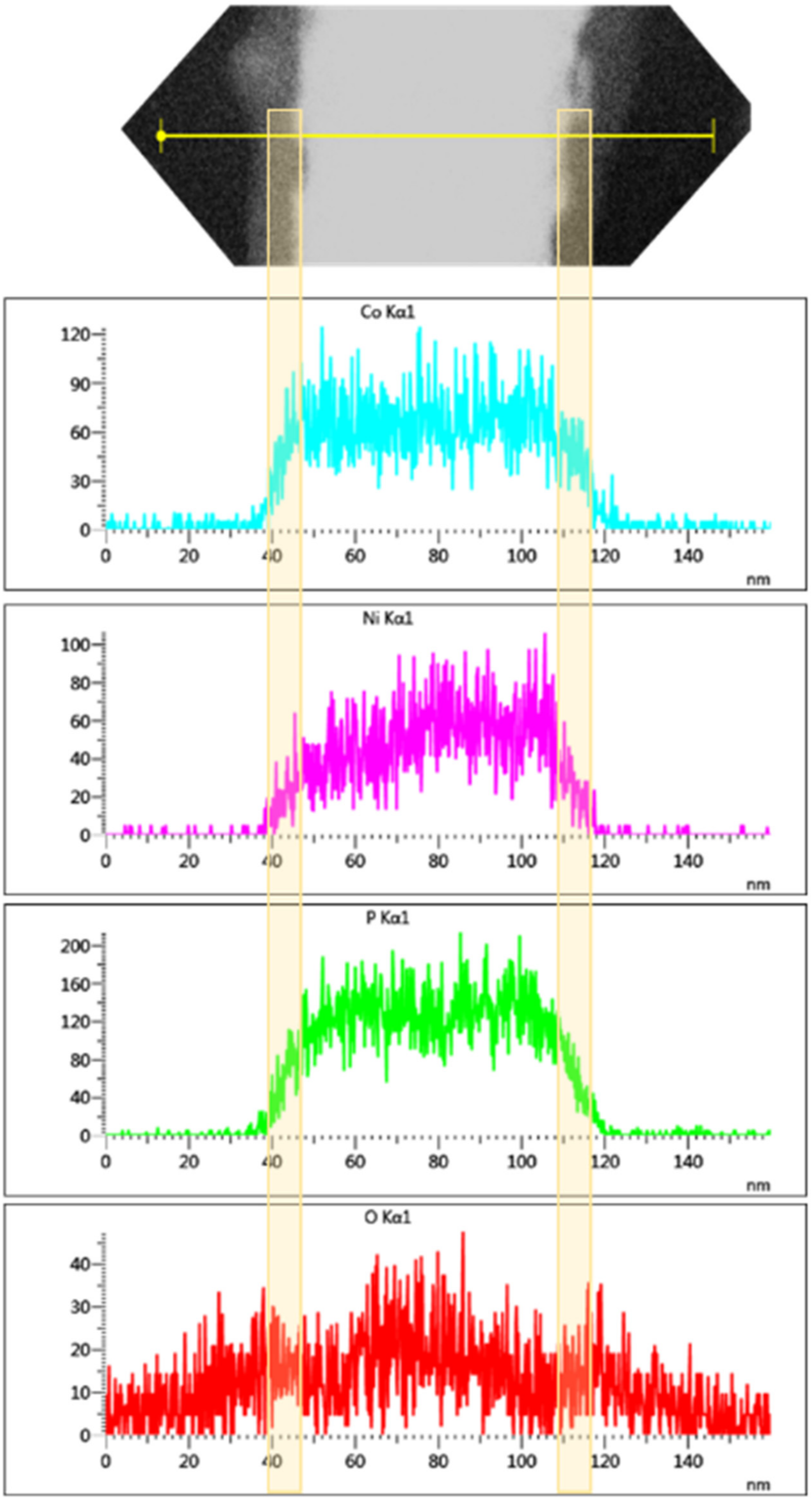

Fig. S7. TEM-EDS line scanning images of different elements in Ni-Co-P/NF.

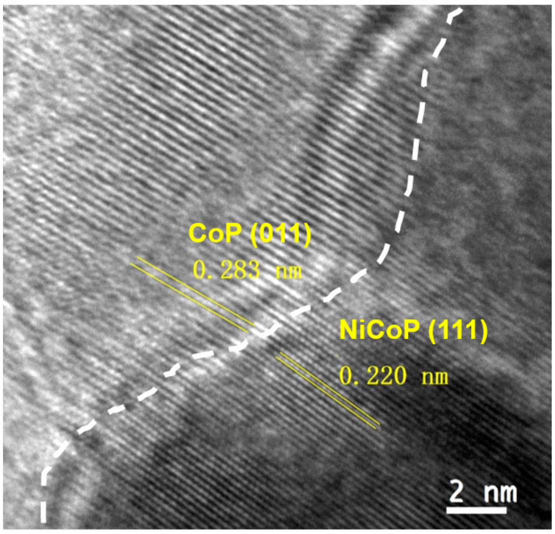

Fig. S8. HRTEM image of Ni-Co-P/NF with interface division.

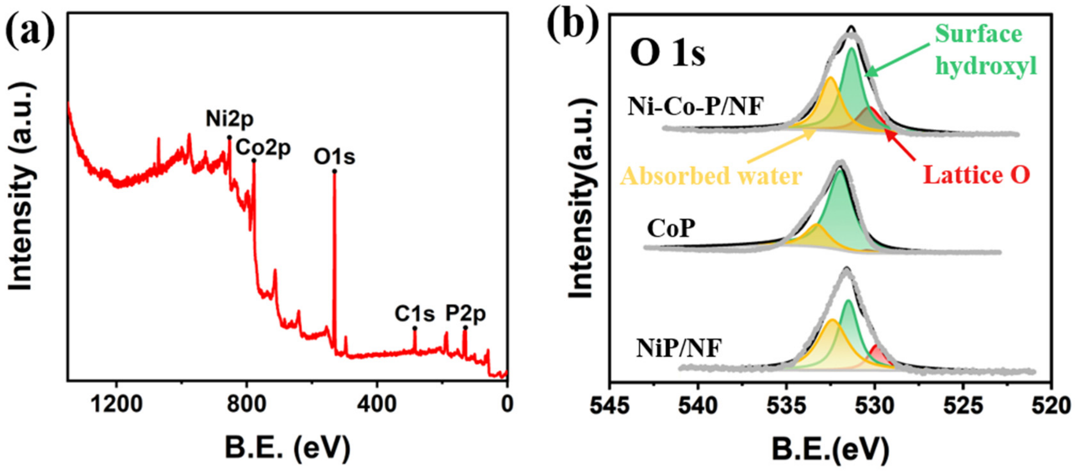

Fig. S9. (a) Survey scan of XPS spectra of the Ni-Co-P/NF; (b) O *1s* of XPS spectra of the Ni-Co-P/NF, CoP, NiP/NF (530.3 eV: the lattice oxygen due to the partial oxidation of the catalyst surface; 531.3 eV: the adsorbed O from surface hydroxyl and adsorbed oxygen species due to the defect of materials; 532.5 eV: surface-absorbed H_2O[1]).

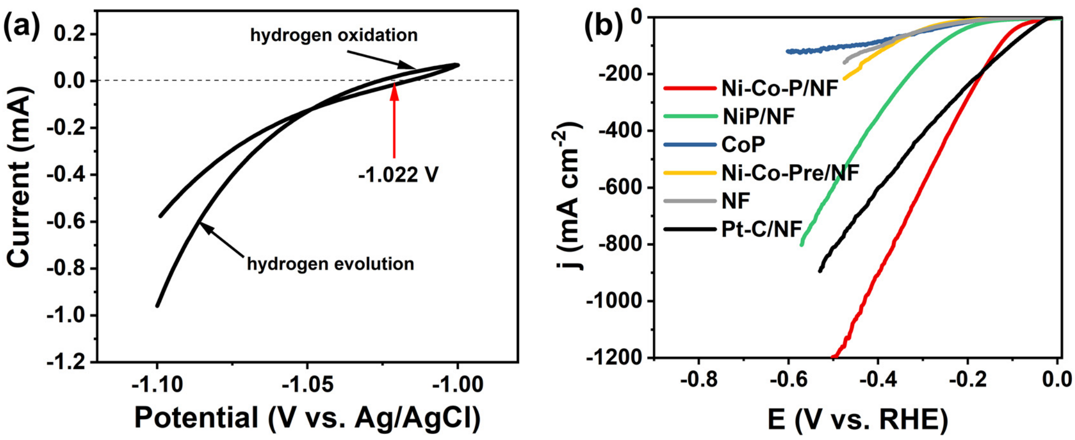

Fig. S10. (a) Potential calibration of the Ag/AgCl reference electrode in 1 M KOH. Potential vs RHE in paper calculated as follows: $E_{RHE}=E_{Ag/AgCl}+1.022V$. (b) The LSV curves without iR compensation at scan rate of 5 mV s^{-1} in 1 M KOH (the overpotentials to reach 10 mA cm^{-2} for Ni-Co-P/NF, NiP/NF, CoP, Ni-Co-Pre/NF and Pt-C/NF are 39 mV, 125 mV, 175 mV, 197 mV and 29 mV, respectively).

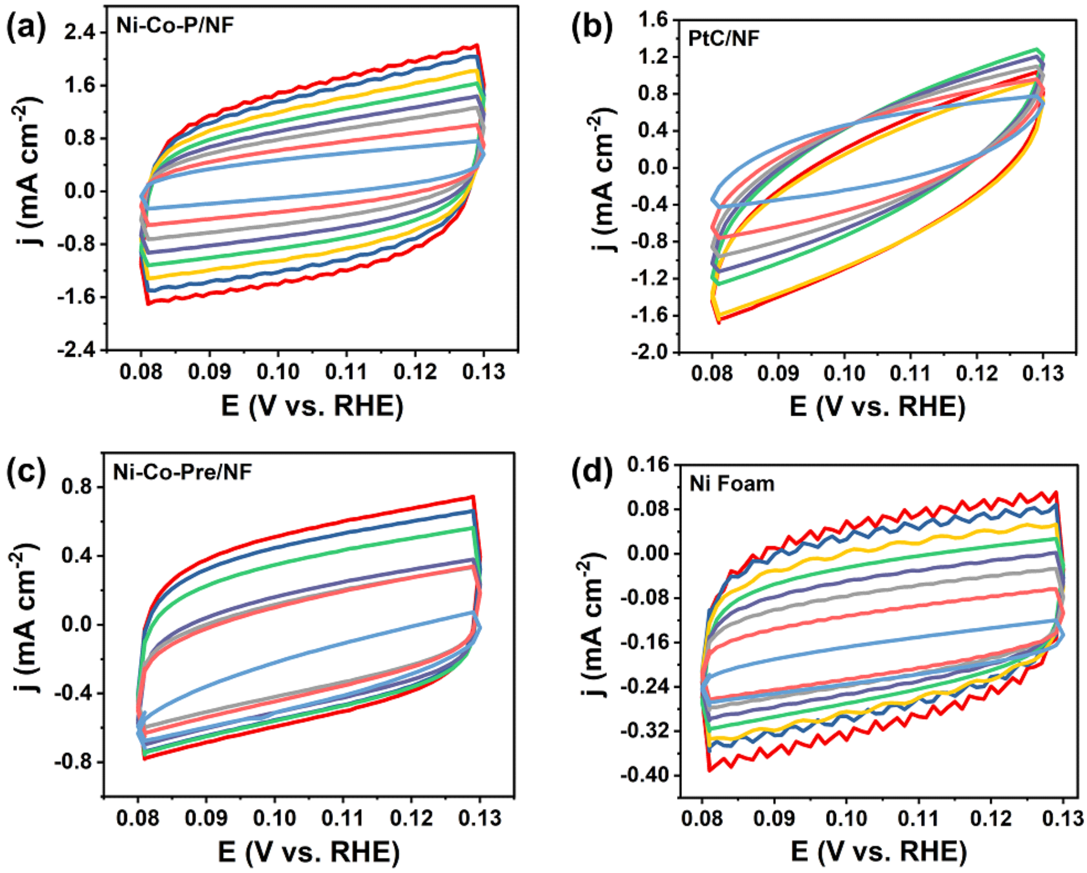

Fig. S11. The CV curves of different samples with the scan rate ranging from 10 to 80 mV s^{-1} in 1.0 M KOH; (a) Ni-Co-P/NF; (b) Pt-C/NF; (c) Ni-Co-Pre/NF; (d) Ni foam.

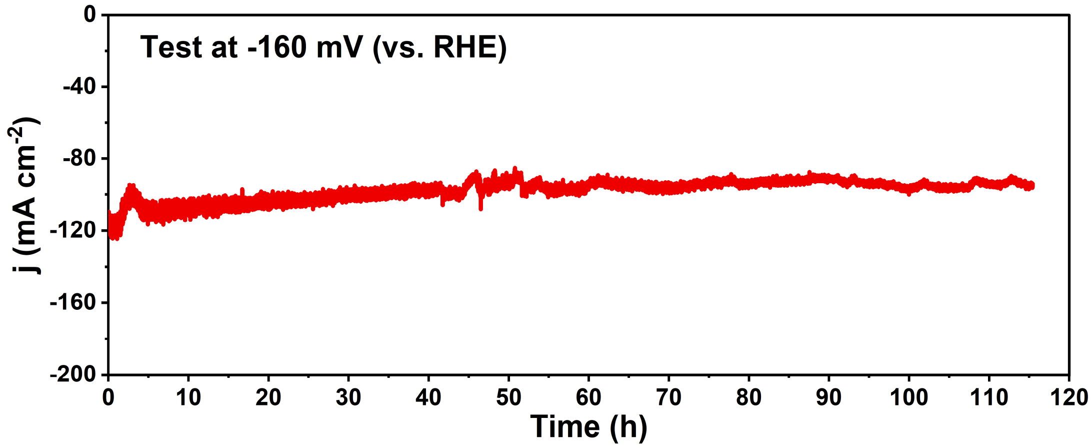

Fig. S12. The stability measurement of Ni-Co-P/NF for HER in 1 M KOH at the constant potential of -160 mV with the current density of about 100 mA cm^{-2}.

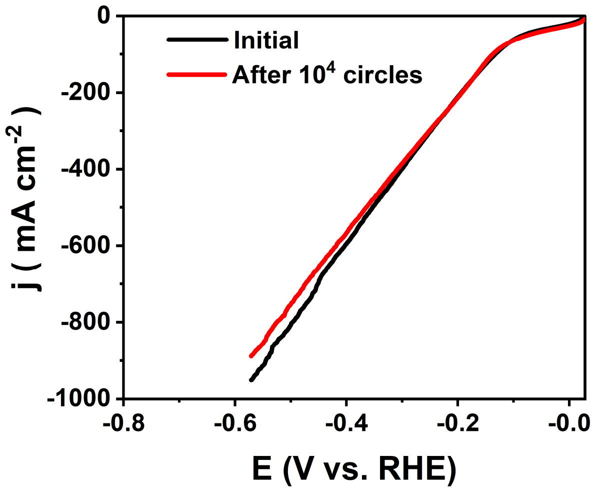

Fig. S13. LSV curves of HER of Ni-Co-P/NF initially and after 10000 cycles without iR compensation.

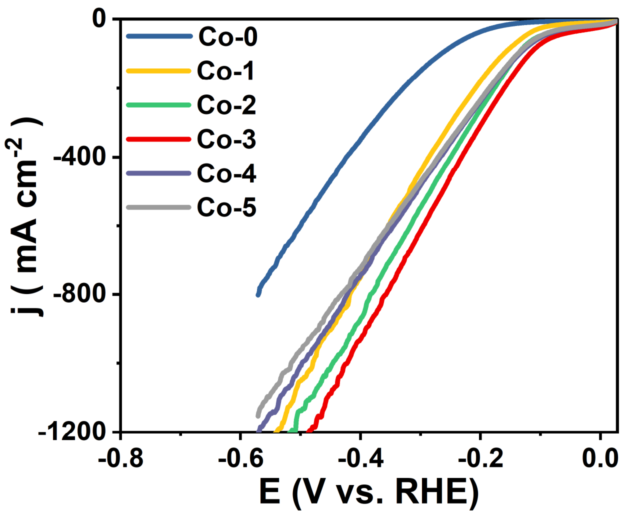

Fig. S14. LSV curves of Ni-Co-P/NF toward HER with different Co addition amounts in precursor without iR compensation.

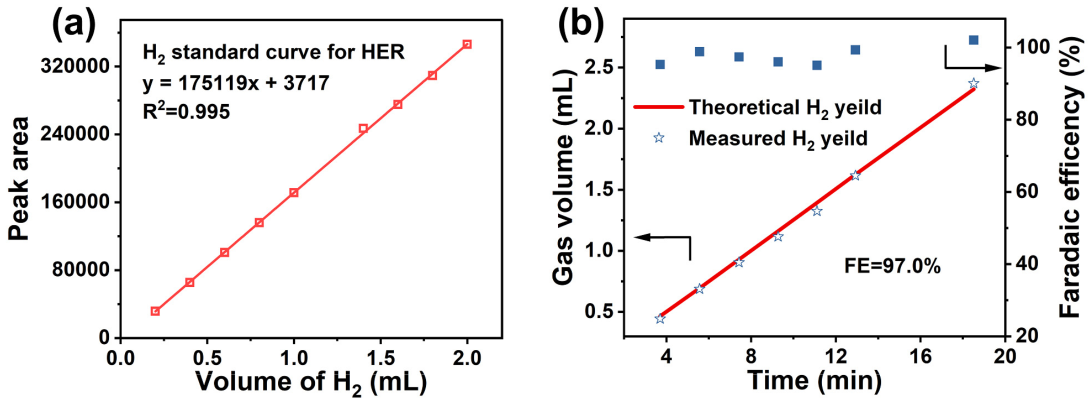

Fig. S15. (a) The calibration curve obtained by plotting volume of H_2 in anode against the peak area of H_2 of GC curve; (b) The Faradaic efficiency of HER with Ni-Co-P/NF in HE unit at fixed potential of 2 V.

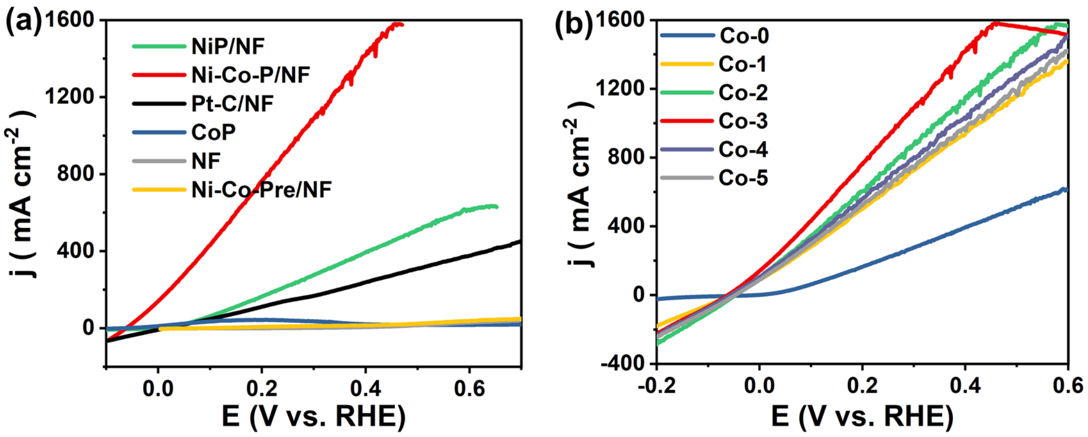

Fig. S16. (a) The LSV curves without iR compensation toward HzOR (Ni-Co-P/NF, NiP/NF, CoP, Ni-Co-Pre/NF and Pt-C/NF require -59 mV, 22 mV, 0 mV, 257 mV and 36 mV to reach 10 mA cm^{-2}, respectively; Ni-Co-P/NF requires -16 mV, 121 mV, 173 mV to reach 100 mA cm^{-2}, 500 mA cm^{-2}, 1000 mA cm^{-2}, respectively). (b) LSV curves of Ni-Co-P/NF toward HzOR with different Co addition amounts in precursor without iR compensation.

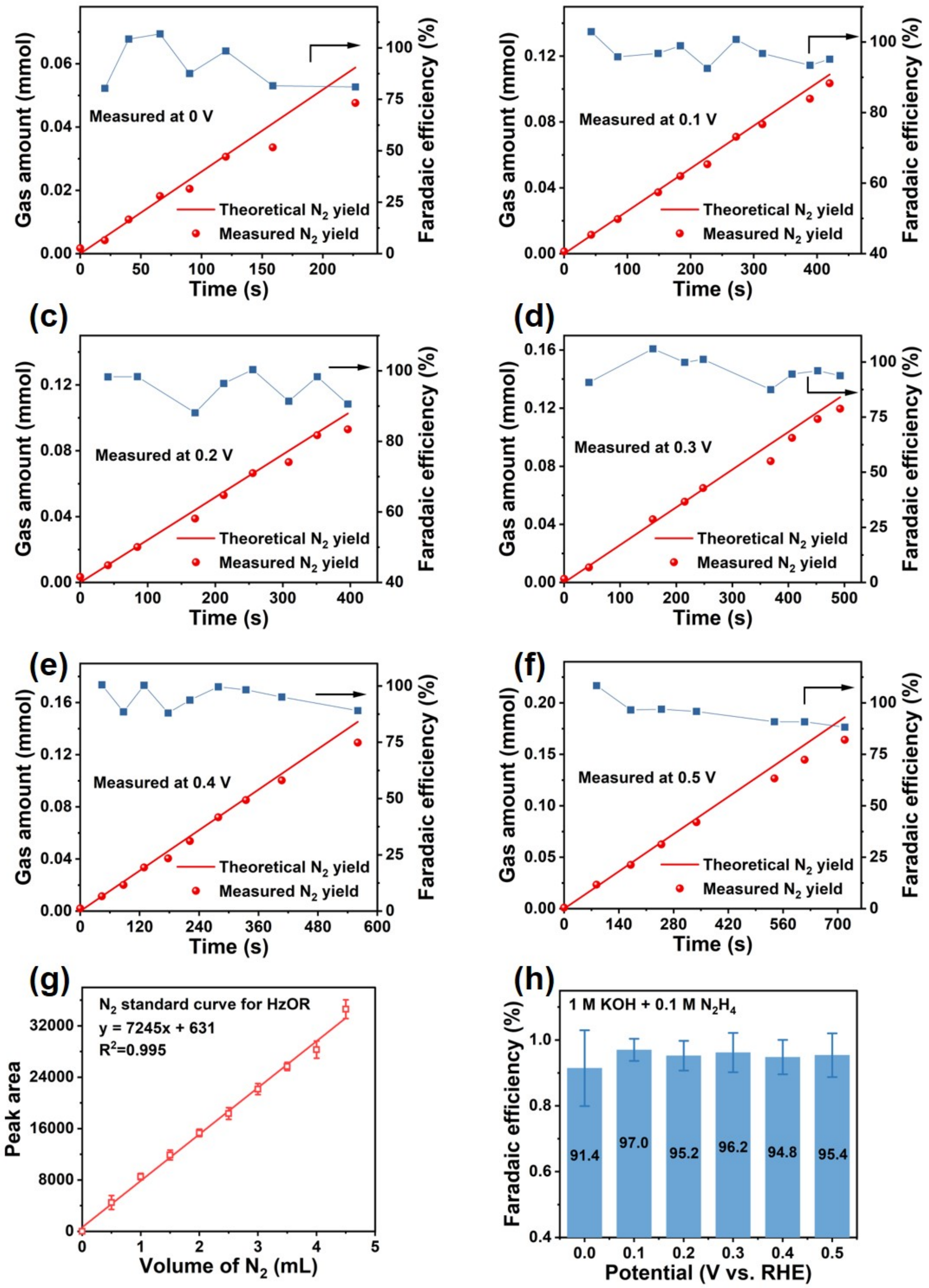

Fig. S17. The Faradaic efficiencies of HzOR with Ni-Co-P/NF in HE unit at multiple potentials. (a) 0 V; (b) 0.1 V; (c) 0.2 V; (d) 0.3 V; (e) 0.4 V; (f) 0.5 V; (g) The calibration curve obtained by plotting volume of N_2 in anode against the peak area of N_2 of GC curve; (g) The average FE of HzOR at different potentials with Ni-Co-P/NF in 1.0 M KOH + 0.1 M N_2H_4. Data are presented as mean ± standard deviation (SD) if N≥3.

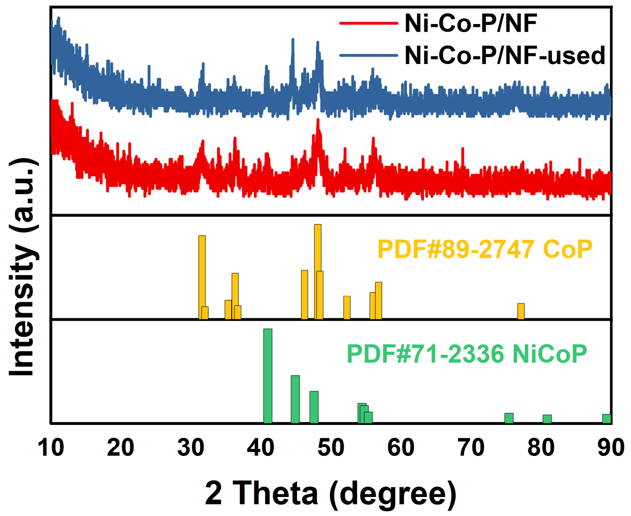

Fig. S18. XRD patterns of Ni-Co-P/NF before and after stability test (named Ni-Co-P/NF-used).

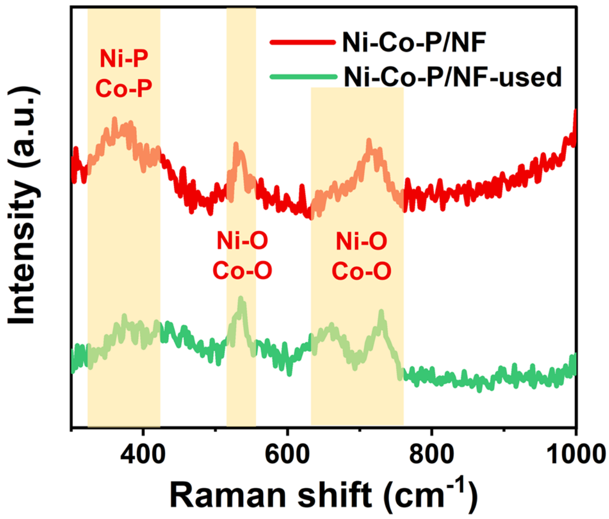

Fig. S19. Raman spectra of Ni-Co-P/NF before and after stability test.

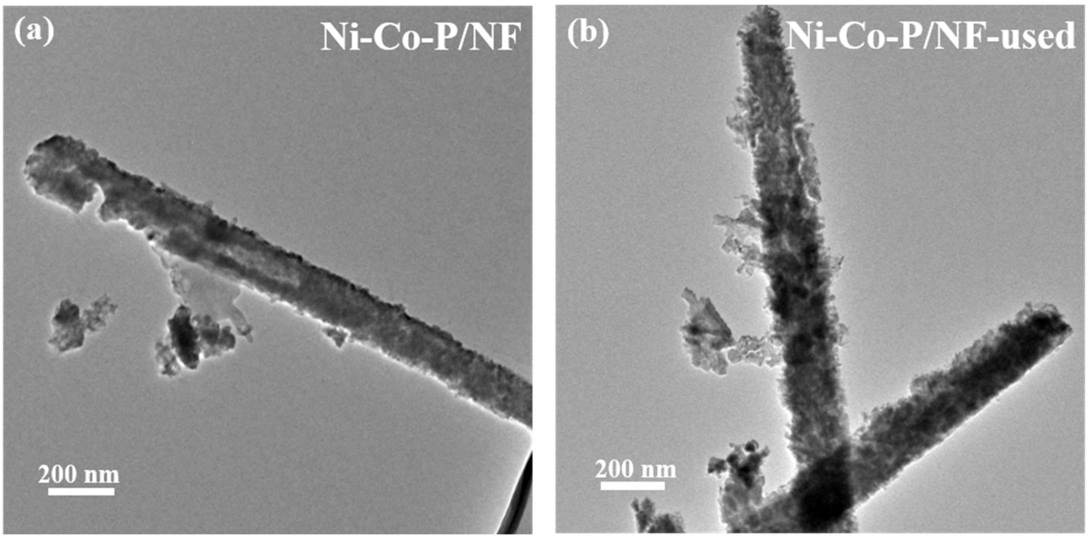

Fig. S20. TEM of Ni-Co-P/NF before and after stability test.

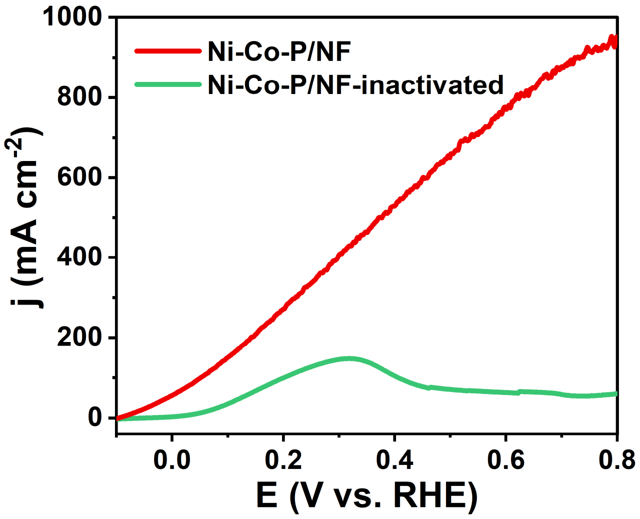

Fig. S21. LSV curves of Ni-Co-P/NF toward HzOR initially and after over-oxidation without iR compensation.

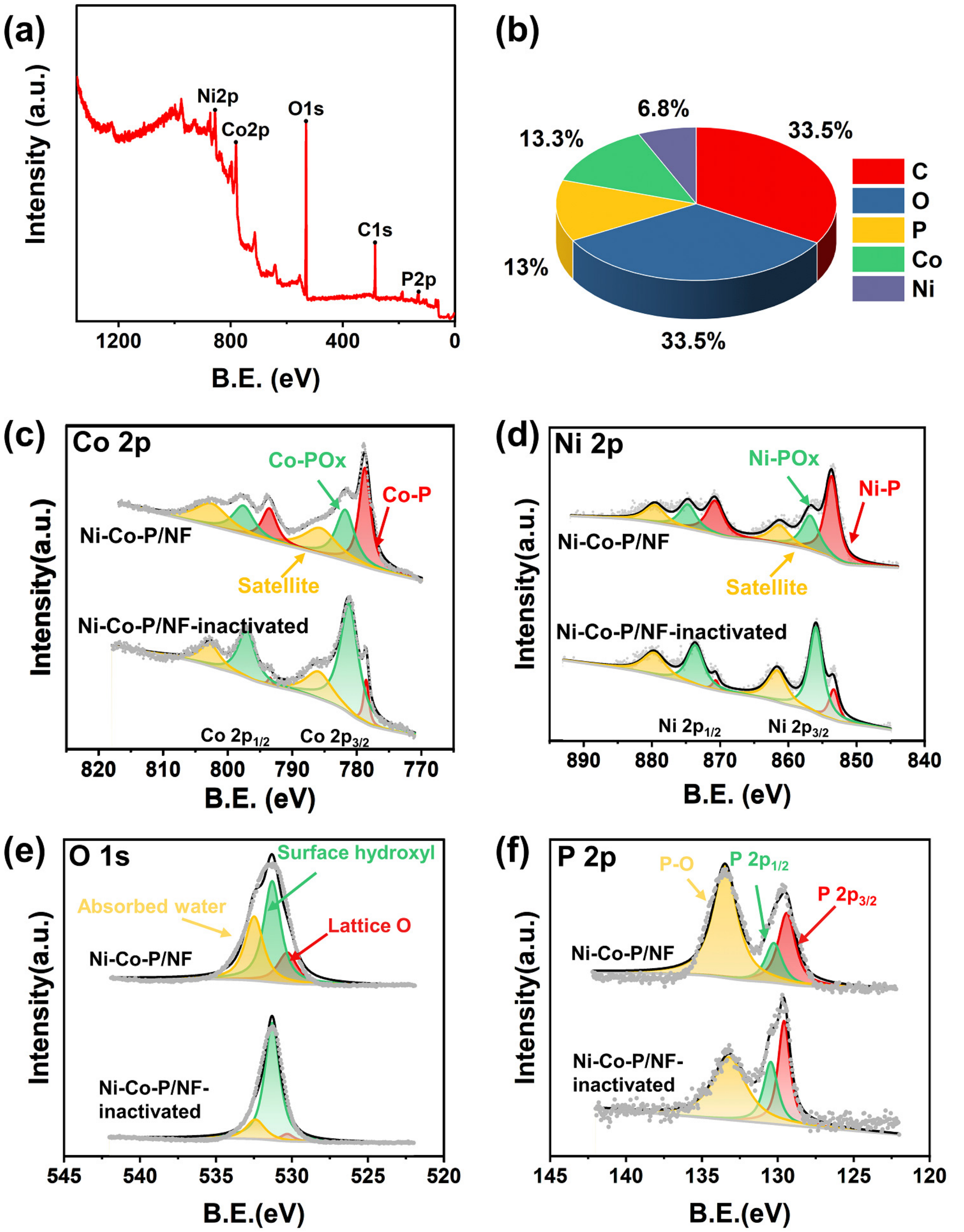

Fig. S22. XPS spectra of Ni-Co-P/NF and inactivated Ni-Co-P/NF-inactivated. (a) Survey scan; (b) Atomic percentage; (c) Co 2p; (d) Ni 2p; (e) O 1s; (f) P 2p.

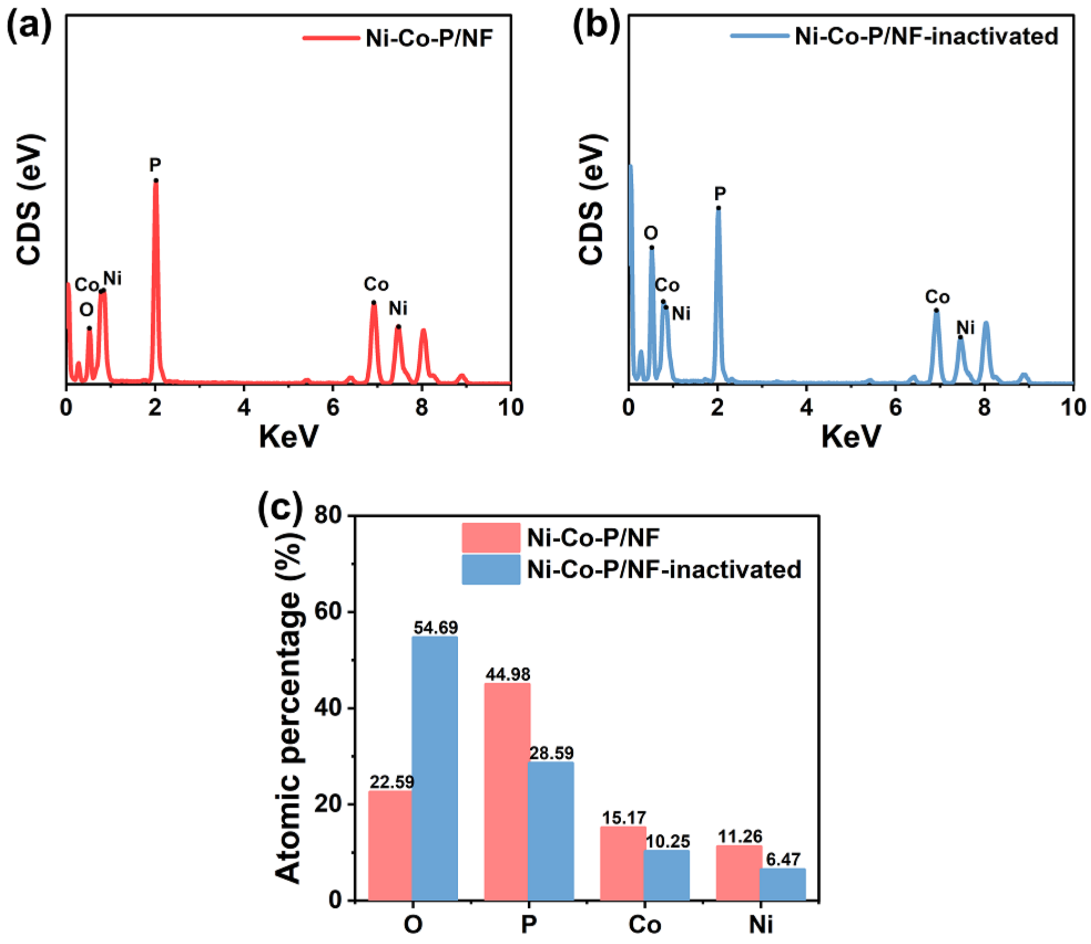

Fig. S23. TEM-EDS images of Ni-Co-P/NF (a) and Ni-Co-P/NF-inactivated (b); (c) the atomic percentages of Ni-Co-P/NF and Ni-Co-P/NF-inactivated.

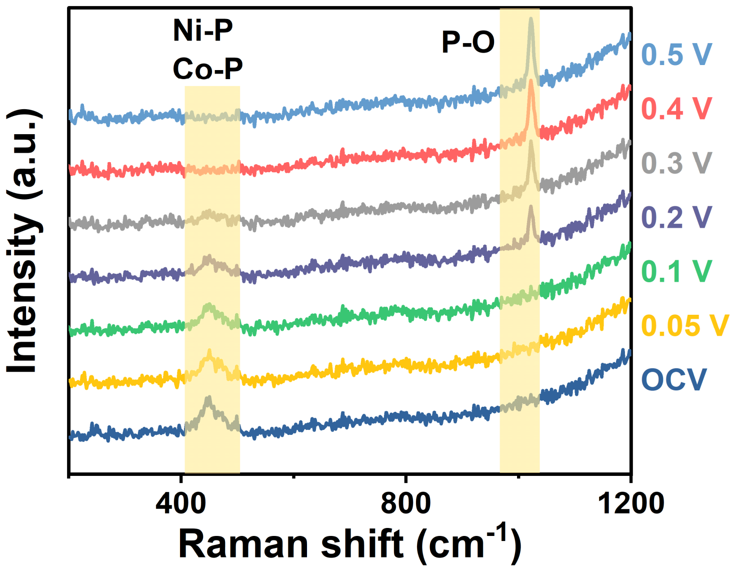

Fig. S24. *In situ* electrochemical Raman spectra of Ni-Co-P/NF in 1 M KOH at varied applied potentials.

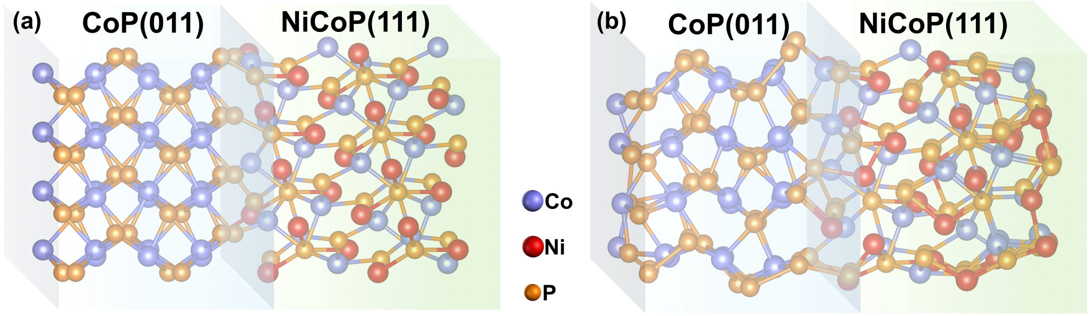

Fig. S25. The structural model to simulate the Ni-Co-P/NF. (a) Initial model; (b) optimized model.

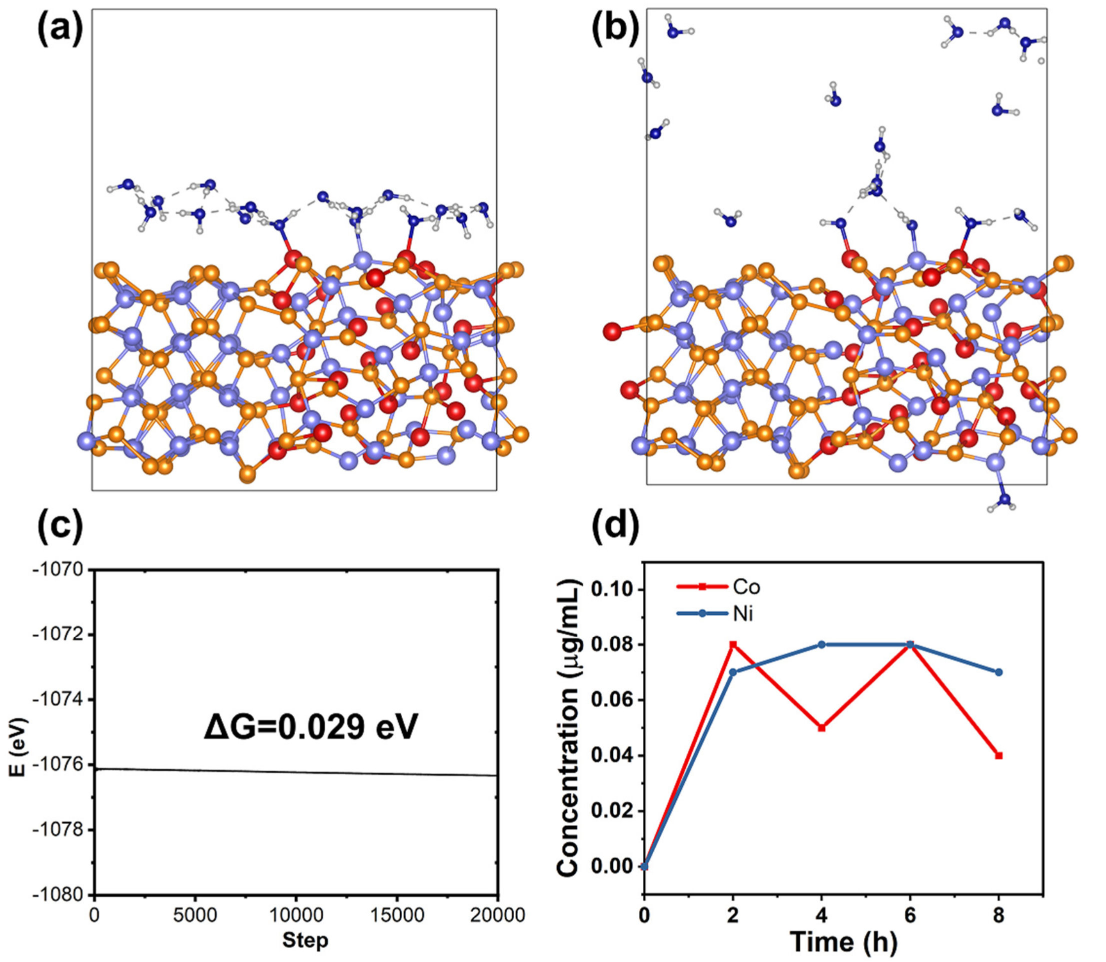

Fig. S26. (a) Ab initio molecular dynamic (AIMD) models to simulate the structure in contact with electrolyte, with a layer of solution (including 15 water molecules and one OH groups) covered on the NiCoP(111)/CoP(011) surface; (b) Model by 20000 steps of kinetic relaxation; (c) AIMD calculation results. (The total energy has a small drift in AIMD calculations, which is caused by the diffusion of water molecules. The overall energy remains highly stable.) (d) Curves of time-dependent concentration of Ni and Co in the electrolyte solution of Ni-Co-P/NF electrode during the i-t test for HzOR.

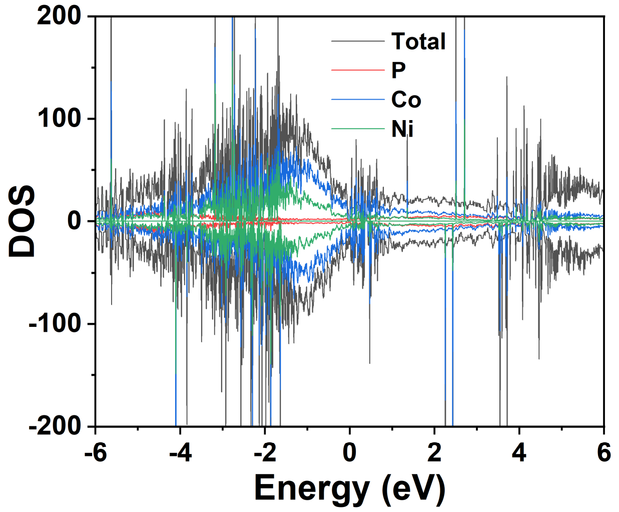

Fig. S27. The density of states (DOS) of Ni, Co, P in NiCoP-CoP. The Fermi level is set at 0 eV.

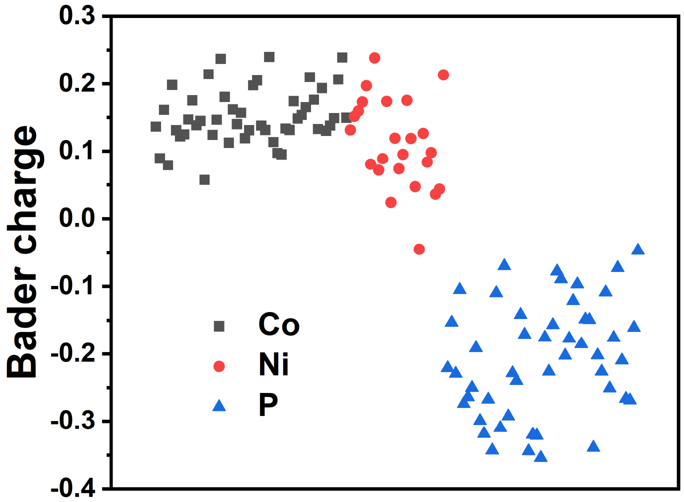

Fig. S28. The Bader charge of Co, Ni, P in NiCoP-CoP.

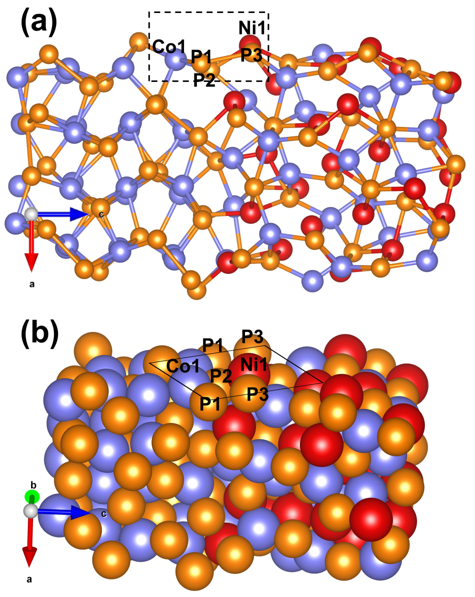

(c)

Adsorption site on CoP(011) | NiCoP(111)	ΔG_{*N2H4} (eV)
Co1	-0.530
Ni1	-0.307
P1	1.289
P2	-----
P3	0.820

Fig. S29. The five different sites at the NiCoP(111)/CoP(011) interface(a, b) and the change of free-energy for N_2H_4 absorption on the sites(c). The ΔG_{*N2H4} on P1, P2 and P3 sites means that the P atom-terminated unit cell is not favorable for N_2H_4 adsorption, which is inconsistent with experimental results.

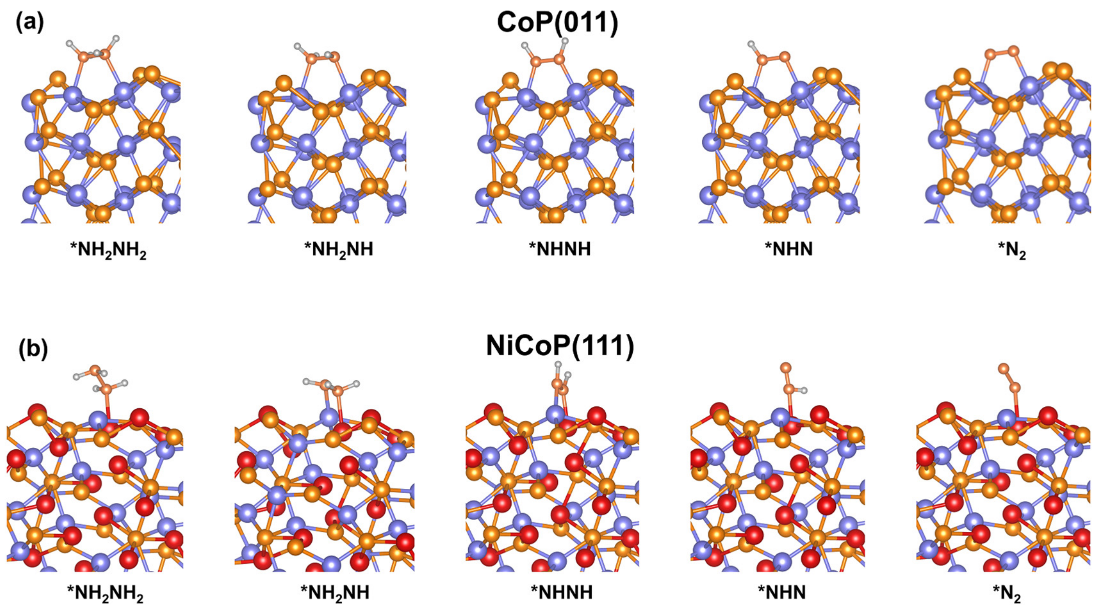

Fig. S30. The models of adsorption intermediates on (a) CoP(011) and (b) NiCoP(111) surfaces.

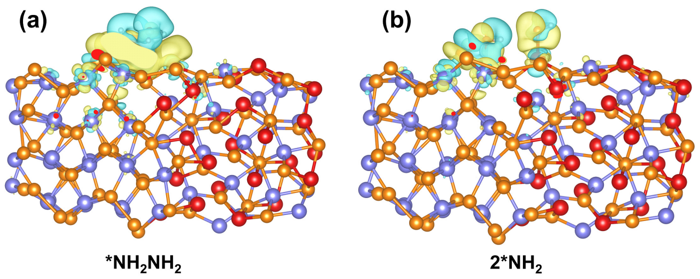

	$L_{N\text{-}N}$ (pm)	$L_{N\text{-}H}$ (pm)
N_2H_4	144.9	102.7
$*N_2H_4$	147.0	102.6
$*NH_2$	(329.3)	101.6

Fig. S31. The charge density difference analysis and the change of bond length of N_2H_4 before and after adsorption at the NiCoP(111)/CoP(011) interface, where the yellow or cyan regions indicate the accumulation or depletion of the charge, respectively.

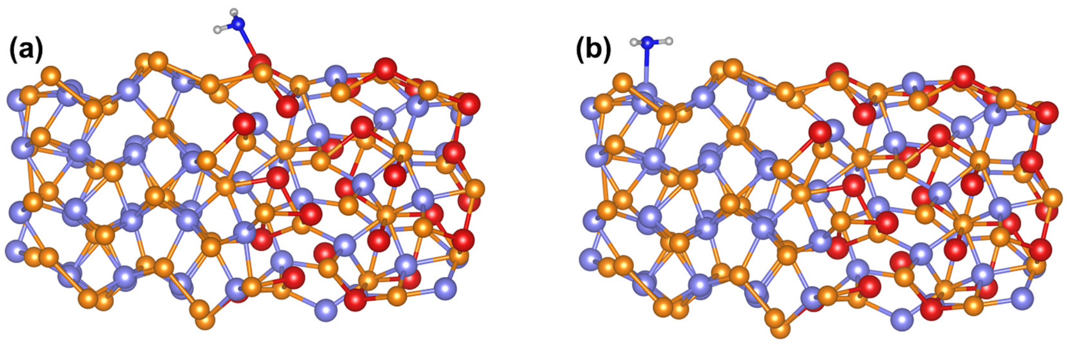

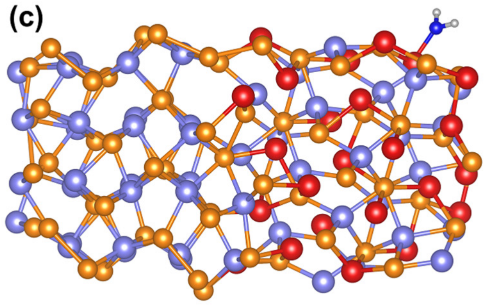

site	ΔG_{*H2O} (eV)
CoP(011) | NiCoP(111)	-0.975
CoP(011)	-0.497
NiCoP(111)	-0.289

Fig. S32. The models of H_2O on (a) NiCoP-CoP site, (b) CoP site, (c) NiCoP site and the change of free-energy for H_2O absorption. The adsorption energy of H_2O at the NiCoP(111)/CoP(011) interface is significantly lower than that on CoP(011) surface(-0.497 eV) and NiCoP(111) surface(-0.289 eV), indicating that the NiCoP-CoP heterostructure has excellent HER kinetics in alkaline electrolyte.

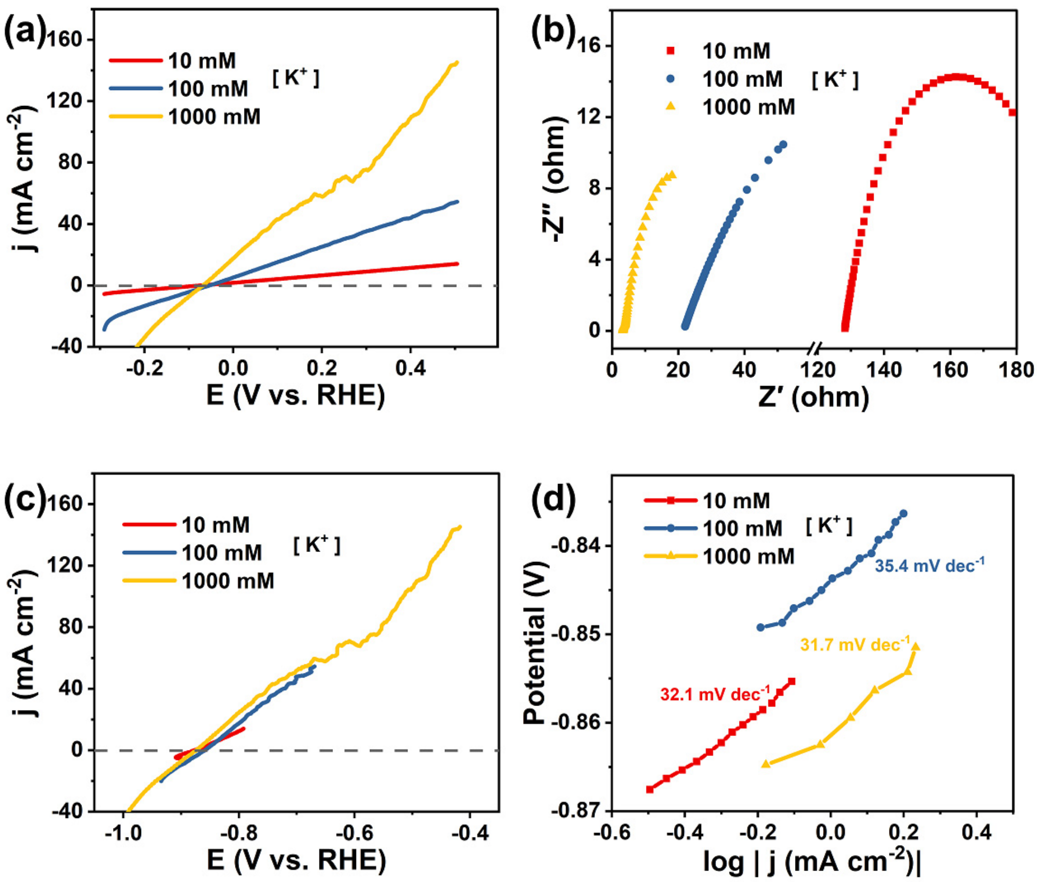

Fig. S33. Electrochemical tests for HzOR on Ni-Co-P/NF surface in 10 mM KOH (pH=12), 10 mM KOH + 90 mM $KClO_4$ (pH=12), 10 mM KOH + 990 mM $KClO_4$ (pH=12). (a) LSV curves without iR compensation; (b) Nyquist plots; (c) LSV curves with iR compensation; (d) Tafel plots after iR compensation.

The K^+ mainly affects the ionic conductivity of the solution (the solution resistance R_s), not the charge-transfer resistance (R_{ct}). The Tafel plots after iR compensation in electrolyte with different K^+ concentrations are almost the same, showing that the influence of K^+ on the reaction kinetics is rather weak, which can be ignored when calculating the energy.

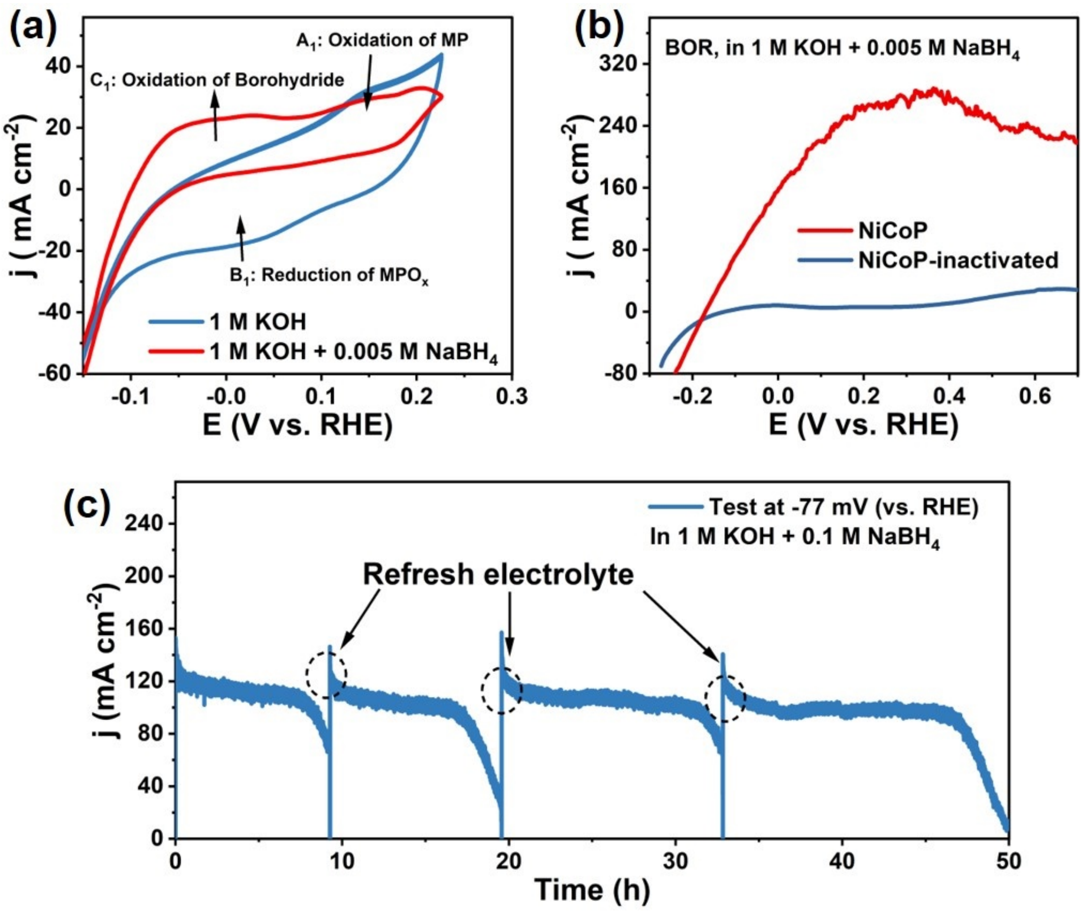

Fig. S34. The mutual promotion relationship between metal phosphide and borohydride. (a) CV curves with or without $NaBH_4$ addition in electrolyte; (b) LSV curves of NiCoP toward BOR initially and after over-oxidation; (c) The stability measurement at about 100 mA cm^{-2} of Ni-Co-P/NF for BOR in 1 M KOH + 0.1 M $NaBH_4$.

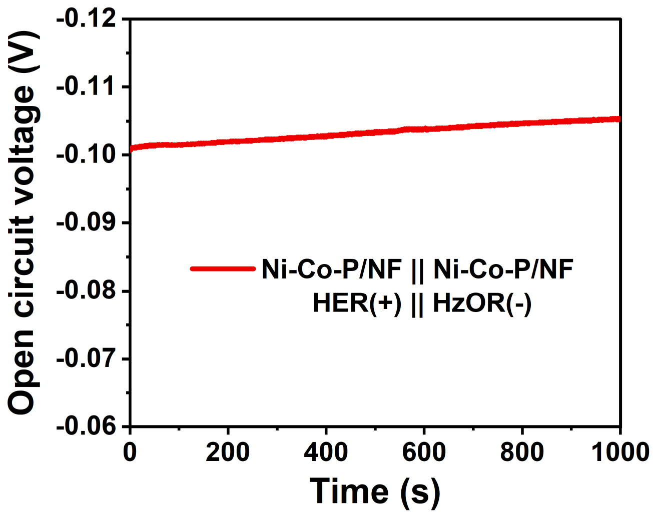

Fig. S35. Open circuit voltage trend over time of the HE unit.

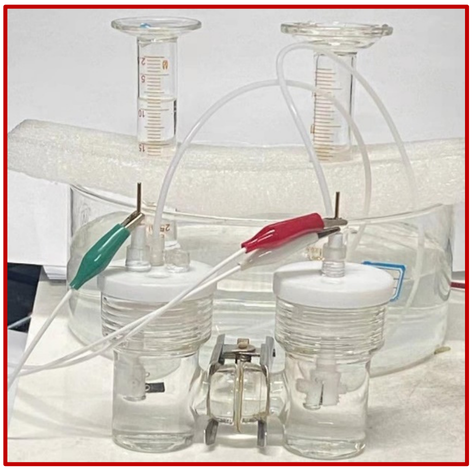

Fig. S36. Schematic diagram of gases collection device for HE unit. The utilization ratio (η) is calculated by $\eta=3V_C/2(V_A+V_C)$; V_A, V_C are the gas volume produced in anode and cathode respectively.

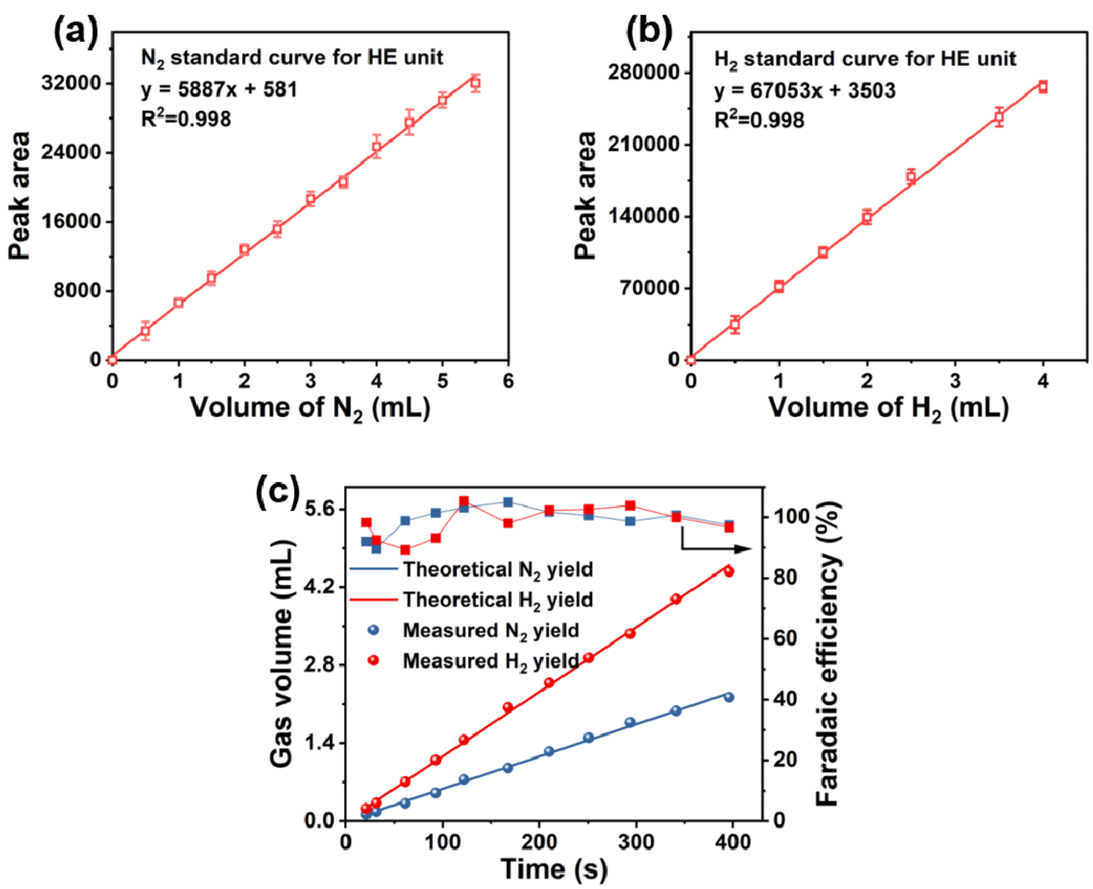

Fig. S37. (a) Calibration curve obtained by plotting volume of N_2 in anode against the peak area of N_2 of GC curve; (b) Calibration curve obtained by plotting volume of H_2 in anode against the peak area of H_2 of GC curve; (c) Faradaic efficiencies of HzOR and HER with Ni-Co-P/NF in HE unit at fixed current of 100 mA cm^{-2}. Data are presented as mean ± standard deviation (SD).

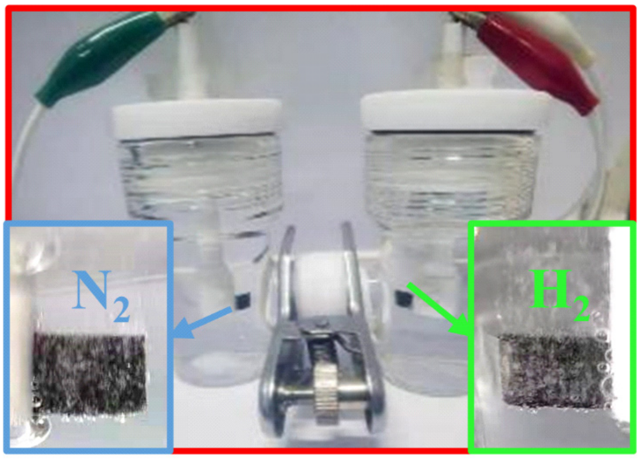

Fig. S38. Digital photograph of the evolution of gas bubbles.

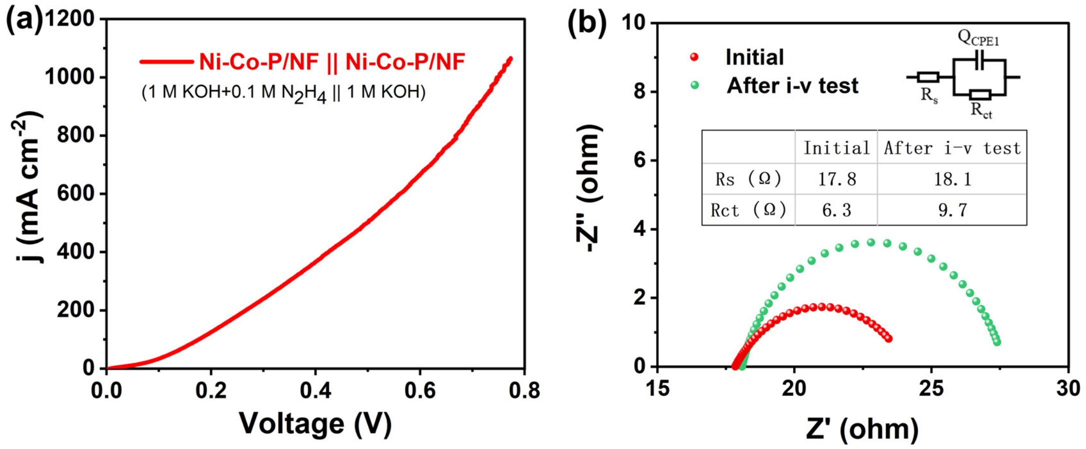

Fig. S39. Electrochemical properties of the HE unit with Ni-Co-P/NF as both anode and cathode catalysts. (a) The 50% iR-corrected LSV curve (The compensation resistance is 9 Ω); (b) Nyquist plots with the fitting pattern in inset.

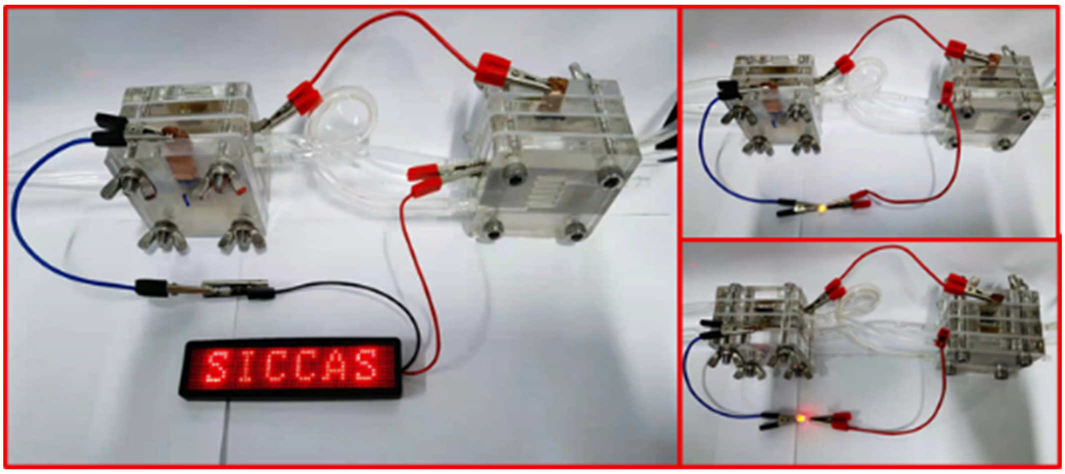

Fig. S40. Digital photograph of DHzFC driving LED.

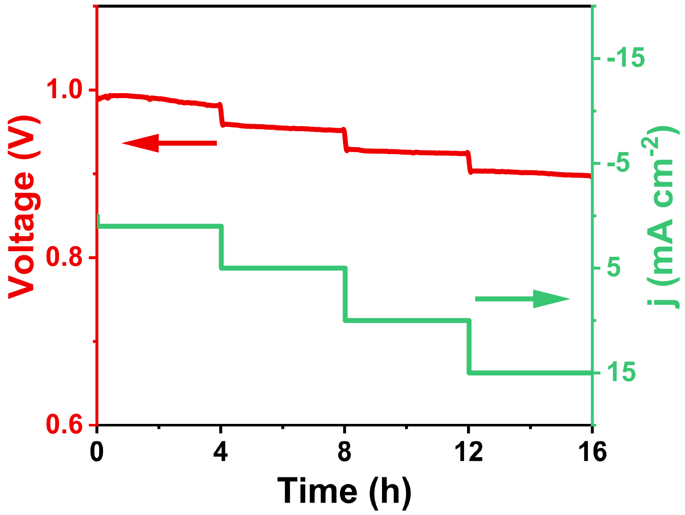

Fig. S41. Stability measurements at different current densities of the DHzFC with Ni-Co-P/NF (+) || Pt-C/NF (-).

Table S1. Comparison of electrocatalytic activities of HER in 1.0 M KOH between Ni-Co-P/NF in this work and various transition metal based catalysts recently reported.

Materials	Current density (mA cm^{-2})	Potential (mV vs. RHE)	Tafel slope (mV dec^{-1})	Stability (h)	Reference
Ni-Co-P/NF	10<br>100<br>500	37<br>115<br>196	33.3	90	This work
NiCo/Mxene	10<br>500	49<br>235	54.2	60	2
Ni_3N-Co_3N/NF	10	43	35.1	40	3
PW-Co_3N/NF	10	41	40	25	4
Fe-CoS_2	10	40	32	40	5
Ni-C HNSA	10	37	31.9	50	6
Ni NCNAs	10	47	89.7	30	7
$CoSe_2$/NF	10	79	84	50	8
Cu_1Ni_2-N/C	10	71.4	106.5	60	9
$NiCo(OH)_x$@ NiCoP/NF	100	164	68.4	20	10
CC@WS2/ Ru-450*	10	32	53.2	100	11
RP-CPM*	10	24	47.3	20	12

*Represents containing precious metal elements

Table S2. EIS parameters of synthesized catalysts for HER in 1 M KOH.

	R_s (Ω)	R_{ct} (Ω)
**Pt-C/NF**	**2.745**	**10.73**
**Ni-Co-P/NF**	**2.904**	**13.09**
**NiP/NF**	**3.178**	**45.9**
**Ni-Co-Pre/NF**	**2.691**	**54.02**
**CoP**	**3.178**	**57.26**

Table S3. EIS parameters of synthesized catalysts for HzOR in 1 M KOH + 0.1 M N_2H_4.

	R_s (Ω)	R_f (Ω)	R_{ct} (Ω)
**Ni-Co-P/NF**	**0.624**	**0.990**	**2.614**
**Pt-C/NF**	**0.836**	**1.564**	**6.162**
**NiP/NF**	**0.893**	**1.538**	**15.36**
**CoP**	**2.772**		**42.25**
**Ni-Co-Pre/NF**	**1.688**		**554.3**

Table S4. Comparison of electrocatalytic activities of HzOR between Ni-Co-P/NF in this work and various transition metal based catalysts recently reported.

Materials	Electrolyte	Current density (mA cm^{-2})	Potential (mV vs. RHE)	Stability （h）	Ref.
Ni-Co-P/NF	1.0 M KOH + 0.1 M N_2H_4	10<br>100<br>1000	-61<br>-24<br>176	100	This work
p-Co/CF	1.0 M KOH + 0.05 M N_2H_4	100	-110	30	13
NiCo/Mxene	1.0 M KOH + 0.5 M N_2H_4	100	-25	30	2
Ni_3N-Co_3N/NF	1.0 M KOH + 0.1 M N_2H_4	100	-9	40	3
PW-Co_3N/NF	1.0 M KOH + 0.1 M N_2H_4	100	-8	10	4
Co_3Ta/C	1.0 M KOH + 0.1 M N_2H_4	100	250	3.33	14
Fe-CoS_2	1.0 M KOH + 0.1 M N_2H_4	100	610	40	5
Ni_2P/NF	1.0 M KOH + 0.1 M N_2H_4	100	-7	10	15
Ni-C HNSA	1.0 M KOH + 0.1 M N_2H_4	10	-20	25	6
Ni NCNAs	1.0 M KOH + 0.3 M N_2H_4	10	-26	24	7
$CoSe_2$/NF	1.0 M KOH + 0.5 M N_2H_4	100	170	14	8
Cu_1Ni_2-N/C	1.0 M KOH + 0.5 M N_2H_4	100	205	35	9
$NiCo(OH)_x$@NiCoP/NF	1.0 M KOH + 0.1 M N_2H_4	100	-40	20	10
Ni_3S_2/NF	1.0 M KOH + 0.2 M N_2H_4	100	415	10	16
CoS_2/TiM	1.0 M KOH + 0.1 M N_2H_4	100	110	10	17
CC@WS2/Ru-450*	1.0 M KOH + 0.5 M N_2H_4	10<br>100	-74<br>-23	100	11
RP-CPM*	1.0 M KOH + 0.3 M N_2H_4	10<br>200	-70<br>82	20	12

*Represents containing precious metal elements.

Table S5. Comparison of hydrazine assisted hydrogen evolution performance of Ni-Co-P/NF and catalysts recently reported as the HER/HzOR electrocatalysts in alkaline condition.

**Materials**	**Electrolyte**	**Current density ($mA\ cm^{-2}$)**	**Cell Voltage (mV)**	**Reference**
**Ni-Co-P/NF (+, –)**	**1.0 M KOH + 0.1 M N_2H_4 || 1.0 M KOH**	**200<br>500**	**240*<br>498***	**This work**
**NiCo@C/MXene/CF (+, –)**	**1.0 M KOH + 0.5 M N_2H_4 (+) || 1.0 M KOH**	**500**	**700***	**2**
**NiFe-LDH (+) || Pt/C (–)**	**0.5 M KOH (+) || 0.5 M NaCl (–)**	**200**	**1600**	**18**
**Ni_3N-Co_3N/NF (+, –)**	**1.0 M KOH + 0.1 M N_2H_4 (+, –)**	**400**	**760***	**3**
**PW-Co_3N/NF (+, –)**	**1.0 M KOH + 0.1 M N_2H_4 (+, –)**	**200**	**227***	**4**
**Fe-CoS_2 (+, –)**	**1.0 M KOH + 0.1 M N_2H_4 (+, –)**	**100**	**610**	**5**
**Ni_2P/NF (+, –)**	**1.0 M KOH + 0.5 M N_2H_4 (+, –)**	**500**	**1000**	**15**

*Represents the data after iR compensation.

1 Liu, H. *et al.* Robust NiCoP/CoP Heterostructures for Highly Efficient Hydrogen Evolution Electrocatalysis in Alkaline Solution. *Acs Applied Materials & Interfaces* **11**, 15528-15536, doi:10.1021/acsami.9b00592 (2019).
2 Sun, F. *et al.* Energy-saving hydrogen production by chlorine-free hybrid seawater splitting coupling hydrazine degradation. *Nature Communications* **12**, 4182, doi:10.1038/s41467-021-24529-3 (2021).
3 Qian, Q. *et al.* Artificial Heterointerfaces Achieve Delicate Reaction Kinetics towards Hydrogen Evolution and Hydrazine Oxidation Catalysis. *Angewandte Chemie International Edition* **60**, 5984-5993, doi:10.1002/anie.202014362 (2021).
4 Liu, Y. *et al.* Manipulating dehydrogenation kinetics through dual-doping Co3N electrode enables highly efficient hydrazine oxidation assisting self-powered H2 production. *Nature Communications* **11**, 1853, doi:10.1038/s41467-020-15563-8 (2020).
5 Liu, X. *et al.* Self-powered H-2 production with bifunctional hydrazine as sole consumable. *Nature Communications* **9**, doi:10.1038/s41467-018-06815-9 (2018).
6 Zhu, Y. *et al.* Dual Nanoislands on Ni/C Hybrid Nanosheet Activate Superior Hydrazine Oxidation-Assisted High-Efficiency H2 Production. *Angewandte Chemie International Edition* **n/a**, doi:10.1002/anie.202113082 (2021).
7 Li, Y. *et al.* Superhydrophilic Ni-based Multicomponent Nanorod-Confined-Nanoflake Array Electrode Achieves Waste-Battery-Driven Hydrogen Evolution and Hydrazine Oxidation. *Small* **17**, doi:10.1002/smll.202008148 (2021).
8 Zhang, J.-Y. *et al.* Anodic Hydrazine Oxidation Assists Energy-Efficient Hydrogen Evolution over a Bifunctional Cobalt Perselenide Nanosheet Electrode. *Angew. Chem.-Int. Edit.* **57**, 7649-7653, doi:10.1002/anie.201803543 (2018).
9 Wang, Z. *et al.* Copper-Nickel Nitride Nanosheets as Efficient Bifunctional Catalysts for Hydrazine-Assisted Electrolytic Hydrogen Production. *Advanced Energy Materials* **9**, doi:10.1002/aenm.201900390 (2019).
10 Li, M. *et al.* 0.03 V Electrolysis Voltage Driven Hydrazine Assisted Hydrogen Generation on NiCo phosphide Nanowires Supported NiCoHydroxide Nanosheets. *Chemelectrochem* **7**, 3089-3097, doi:10.1002/celc.202000604 (2020).
11 Li, J. *et al.* Elucidating the Critical Role of Ruthenium Single Atom Sites in Water Dissociation and Dehydrogenation Behaviors for Robust Hydrazine Oxidation-Boosted Alkaline Hydrogen Evolution. *Advanced Functional Materials* **32**, doi:10.1002/adfm.202109439 (2022).
12 Li, Y. *et al.* Partially exposed RuP2 surface in hybrid structure endows its bifunctionality for hydrazine oxidation and hydrogen evolution catalysis. *Science Advances* **6**, doi:10.1126/sciadv.abb4197 (2020).
13 Liu, Q. *et al.* Low-coordinated cobalt arrays for efficient hydrazine electrooxidation. *Energy & Environmental Science*, doi:10.1039/d2ee01463g (2022).
14 Feng, G. *et al.* Atomically ordered non-precious Co3Ta intermetallic nanoparticles as high-performance catalysts for hydrazine electrooxidation. *Nature Communications* **10**, doi:10.1038/s41467-019-12509-7 (2019).
15 Tang, C. *et al.* Energy-Saving Electrolytic Hydrogen Generation: Ni2P Nanoarray as a High-Performance Non-Noble-Metal Electrocatalyst. *Angewandte Chemie International Edition* **56**, 842-846, doi:10.1002/anie.201608899 (2017).
16 Liu, G. *et al.* Vapor-phase hydrothermal transformation of a nanosheet array structure Ni(OH)(2)

into ultrathin Ni3S2 nanosheets on nickel foam for high-efficiency overall water splitting. *Journal of Materials Chemistry A* **6**, 19201-19209, doi:10.1039/c8ta07162d (2018).

17 Ma, X. *et al.* Hydrazine-assisted electrolytic hydrogen production: CoS2 nanoarray as a superior bifunctional electrocatalyst. *New Journal of Chemistry* **41**, 4754-4757, doi:10.1039/c7nj00326a (2017).

18 Dresp, S. *et al.* Efficient direct seawater electrolysers using selective alkaline NiFe-LDH as OER catalyst in asymmetric electrolyte feeds. *Energy & Environmental Science* **13**, 1725-1729, doi:10.1039/d0ee01125h (2020).
