# Supplementary material for: Active site recovery and N-N bond breakage during hydrazine oxidation boosting the electrochemical hydrogen production
Source: Nat Commun. 2023 Apr 10;14:1997. doi: 10.1038/s41467-023-37618-2 (PMC10083172; doi:10.1038/s41467-023-37618-2)
Supplement: Supplementary file 3 — Description to Additional Supplementary Information [file 41467_2023_37618_MOESM3_ESM.pdf]

Description of Additional Supplementary files

Supplementary Movie. The working video of self-powered hydrogen production system, with the HE unit powered by DHzFC.
